# The Sum of Tumour-to-Brain Ratios Improves the Accuracy of Diagnosing Gliomas Using 18F-FET PET

**DOI:** 10.1371/journal.pone.0140917

**Published:** 2015-10-15

**Authors:** Bogdan Malkowski, Maciej Harat, Agnieszka Zyromska, Tomasz Wisniewski, Aleksandra Harat, Rita Lopatto, Jacek Furtak

**Affiliations:** 1 Department of Positron Emission Tomography and Molecular Imaging, Nicolaus Copernicus University, Ludwik Rydygier Collegium Medicum, Bydgoszcz, Poland; 2 Department of Radiotherapy, Franciszek Lukaszczyk Oncology Centre, Bydgoszcz, Poland; 3 Department of Public Health, Nicolaus Copernicus University, Ludwik Rydygier Collegium Medicum, Bydgoszcz, Poland; 4 Department of Oncology and Brachytherapy, Nicolaus Copernicus University, Ludwik Rydygier Collegium Medicum, Bydgoszcz, Poland; 5 Department of Neurosurgery, 10th Military Research Hospital and Polyclinic, Bydgoszcz, Poland; 6 Department of Nuclear Medicine, Franciszek Lukaszczyk Oncology Centre, Bydgoszcz, Poland; The George Washington University, UNITED STATES

## Abstract

Gliomas are common brain tumours, but obtaining tissue for definitive diagnosis can be difficult. There is, therefore, interest in the use of non-invasive methods to diagnose and grade the disease. Although positron emission tomography (PET) with 18F-fluorethyltyrosine (18F-FET) can be used to differentiate between low-grade (LGG) and high-grade (HGG) gliomas, the optimal parameters to measure and their cut-points have yet to be established. We therefore assessed the value of single and dual time-point acquisition of 18F-FET PET parameters to differentiate between primary LGGs (n = 22) and HGGs (n = 24). PET examination was considered positive for glioma if the metabolic activity was 1.6-times higher than that of background (contralateral) brain, and maximum tissue-brain ratios (TBR_max_) were calculated 10 and 60 min after isotope administration with their sums and differences calculated from individual time-point values. Using a threshold-based method, the overall sensitivity of PET was 97%. Several analysed parameters were significantly different between LGGs and HGGs. However, in a receiver operating characteristics analysis, TBR sum had the best diagnostic accuracy of 87% and sensitivity, specificity, and positive and negative predictive values of 100%, 72.7%, 80%, and 100%, respectively. 18F-FET PET is valuable for the non-invasive determination of glioma grade, especially when dual time-point metrics are used. TBR sum shows the greatest accuracy, sensitivity, and negative predictive value for tumour grade differentiation and is a simple method to implement. However, the cut-off may differ between institutions and calibration strategies would be useful.

## Introduction

Gliomas account for 80% of all primary tumours of the central nervous system (CNS) and can be associated with poor clinical outcomes [1]. They are often unresectable, and they are biologically and histologically heterogeneous. Improved and reliable diagnostic tools are required to improve patient stratification to match them with the most effective adjuvant therapy, including radiotherapy and chemotherapy. Since about 50% of brain tumours cannot be totally removed and subjected to complete histological assessment [2], there is great interest in the development and use of non-invasive methods that accurately reveal the biological status of the disease. Such methods are also extremely important for obtaining image-guided biopsies to provide tissue for histological diagnosis, especially since biopsy-based diagnosis differs from resection-based diagnosis in up to 50% of cases [2].

Positron emission tomography (PET) using the amino acid analogue 18F-fluorethyltyrosine (18F-FET) has been shown to reliably reflect glioma tissue properties, including differences between low-grade (LGG) and high-grade (HGG) tumours [3]. For CNS diagnostics, 18F-FET has advantages over other radiotracers due to its high stability *in vitro* and *in vivo*, fast tumour kinetics, low susceptibility to accumulation in healthy tissues, straightforward synthesis, and long half-life [4–7]. 18F-FET PET is a proven tool in the differential diagnosis of malignant and benign brain tumours [8,9], with PET-guided biopsies confirming the technique’s diagnostic efficacy for the detection of the solid components of gliomas and their microscopic infiltration [10]. Various parameters describing 18F-FET uptake in a tumour mass have been published. However, studies on the usefulness of 18F-FET PET in initial brain tumour grading and histological mapping are still scarce and based on results from only a limited number of patients. The reliability of such estimations and the optimal methodology to use are still a matter of controversy and have yet to be standardised. The current study assesses the value of both single and dual time-point acquisition of 18F-FET PET parameters to differentiate between primary low-grade gliomas (LGGs) and high-grade gliomas (HGGs) to improve 18F-FET PET reproducibility between different facilities.

## Materials and Methods

### Study design

This retrospective study was conducted in the Franciszek Lukaszczyk Oncology Centre in Bydgoszcz, Poland. Consecutive patients diagnosed at our institution between February 2009 and December 2013 that met the following eligibility criteria were included: a suspicion of primary brain glioma based on routine magnetic resonance imaging (MRI), histopathological confirmation of the diagnosis, no previous treatment, preoperative 18F-FET PET, and subsequent surgery of at least subtotal resection conducted in the 12 months after PET. Forty-six patients (mean age 46 years, range 24–71; 24 men, 22 women) were eligible for study, in whom 22 (47.48%) low-grade and 24 (52.17%) high-grade gliomas were diagnosed (Table 1).

**Table 1:** Histopathological diagnoses of the tumours examined in this study.

Pathology	Number of patients (%) Total number = 46 (100%)
**Low-grade gliomas (LGGs)**	**22 (47.83)**
Astrocytoma optic neuroglioma	1 (2.17)
Astrocytoma fibrillare	11 (23.91)
Ganglioglioma	1 (2.17)
Astrocytoma gemistocyticum	2 (4.35)
Oligoastrocytoma	6 (13.04)
Oligodendroglioma	1 (2.17)
**High-grade gliomas (HGGs)**	**24 (52.17)**
Oligoastrocytoma anaplasticum	2 (4.35)
Astrocytoma anaplasticum	9 (19.56)
Glioblastoma multiforme	13 (28.26)

### Ethical statement

The study was performed in accordance with the principles of the Helsinki Declaration and was approved by the Ethics Committee of Collegium Medicum of Nicolaus Copernicus University. All patients gave written informed consent before each 18F-FET PET investigation. Patients were investigated twice using single and dual time-point 18F-FET PET examinations.

### PET imaging and data analysis

Patients fasted for at least four hours before administration of ^18^F-FET tracer to maintain similar test conditions. The examinations were performed using a Biograph mCT128 scanner (Siemens AG, Berlin, Germany). All participants underwent head imaging 10 and 60 min after injection with 18F-FET. Patients received 350 ± 10 MBq of tracer intravenously. The acquisition of the head was performed with the patient’s arms placed alongside the body.

A CT scan was acquired during shallow breathing with the following parameters: Care Dose 4D, 120 kV, pitch 0.7. The PET scan was acquired with acquisition times of 2.7 min per bed position. CT data were used for attenuation correction. Images were reconstructed using a commercial three-dimensional iterative reconstruction algorithm called TrueX+tof (UltraHD-PET) (matrix 200×200, interval 3 mm, 2 iterations, 21 subsets).

18F-FET tissue uptake was expressed using the standardised uptake value (SUV), dividing the radioactivity (MBq/ml) in the tissue by the radioactivity injected per gram of body weight. The maximal standardised uptake value (SUV_max_) for each lesion was calculated on PET images with the CT image as the reference. To differentiate between the metabolic activity of ^18^F-FET in gliomatous areas and unaffected regions, a volume of interest (VOI) was created in the symmetrical contralateral hemisphere, in which the physiological activity of the tissue was denoted “background activity” (BG). VOIs were always similar in size, shape, and localisation to the contralateral tumour area and included grey and white matter.

PET examination was considered positive for glioma if the metabolic activity was 1.6-times higher than that of background [4]. Biological tumour volume (BTV) was defined by the assessment of increased 18F-FET uptake in a semi-automatic threshold-based (SUV_max_/BG≥1.6) delineation of the VOI. Based on the maximal uptake in a tumour divided by the maximal uptake in the normal contralateral hemisphere brain tissue (SUV_max_/BG_max_), a maximum tissue-to-brain ratio (TBR_max_) parameter was defined.

Two nuclear medicine specialists with experience working with ^18^F-FET tracer and an experienced radiologist evaluated each case.

### Single time-point parameters of PET evaluation

SUV10 and SUV60 were defined based on SUV_max_ measured up to 10 and 60 min after 18F-FET tracer injection, respectively. The maximum tissue-brain ratio (TBR_max_) was also calculated for the chosen time intervals (TBR10 and TBR60).

### Dual time-point parameters of PET evaluation

An increase or decrease in SUVmax between time points of an acquisition were determined by the SUV and TBR difference (SUV60-SUV10 and TBR60-TBR10), relative values (SUV60/SUV10 and TBR60/TBR10), and sums (SUV60+SUV10 and TBR60+TBR10).

### Statistical analysis

Since variables were not normally distributed, the non-parametric Mann—Whitney U-test was used to assess differences between groups. Parameter values were reported as the median (Me), lower quartile (Q1), and upper quartile (Q3). The sensitivity, specificity, and positive/negative predictive values of the parameters and cut-off values were assessed using receiver operating characteristics (ROC) curves. The PET parameter with greatest predictive value was chosen using the area under the ROC curve (AUC). The AUC value reflected the diagnostic accuracy: AUC: 0.9–1.0 –very good; 0.8–0.9 –good; 0.7–0.8 –satisfactory; 0.6–0.7 –fairly good; 0.5–0.6 –inadequate. P values of less than 0.05 were considered significant. Statistical analyses were performed using STATISTICA software (StatSoft^®^ version 10.0 PL, StatSoft Inc., Tulsa, OK) and Medical Set software (Site Licence).

## Results

Of 46 cases, one FET-PET result was negative before surgery. Subsequent pathological evaluation of the resected tumour confirmed astrocytoma diffusum, WHO II. The median values of PET parameters acquired at single and dual time-points in HGG and LGG patients undergoing at least subtotal surgery are shown in Table 2. Mean BTV values were 16 and 14 mL for HGGs and LGGs, respectively. Median values of several analysed parameters (SUV10, SUV60, TBR10, TBR60, relative SUV and TBR, SUV sum and difference, and TBR sum and difference) were significantly different between LGGs and HGGs (Table 2).

**Table 2:** Values of single and dual time-point PET parameters for high-grade and low-grade gliomas.

Parameter	Low-grade gliomas (n = 22)	High-grade gliomas (n = 24)	p-value
SUV10	1.89 (1.56–2.45)	3.24 (2.65–4.13)	<0.001
SUV60	2.27 (2.01–2.65)	2.99 (2.58–3.74)	0.002
TBR10	1.24 (1.01–1.84)	2.74 (2.04–3.23)	<0.001
TBR60	1.39 (1.07–1.77)	2.25 (1.91–2.47)	<0.001
Relative SUV	1.11 (0.99–1.32)	0.91 (0.90–1.08)	0.013
Relative TBR	1.01 (0.95–1.11)	0.83 (0.72–1.00)	0.017
SUV difference	0.26 (0.00–0.50)	-0.30 (-0.42–0.23)	0.01
TBR difference	0.03 (-0.05–0.14)	-0.49 (-0.88–0.01)	0.001
SUV sum	4.12 (3.64–4.76)	5.89 (5.45–7.31)	<0.001
TBR sum	2.65 (2.16–3.56)	5.19 (3.96–5.67)	<0.001

We next used ROC curves to choose the optimal parameter(s) to differentiate between LGGs and HGGs. TBR sum had the greatest AUC (0.852), with a value of 87% reflecting good diagnostic accuracy. The sensitivity, specificity, and positive and negative predictive values were 100%, 72.7%, 80%, and 100%, respectively. In the single time-point evaluation, TBR10 achieved the highest accuracy (82.6%), as shown in Table 3. The ROC analysis indicated that a TBR10 threshold of at least 1.44 and a TBR sum threshold of at least 3.15 best differentiated HGGs and LGGs.

**Table 3:** Parameter values as predictive factors for high-grade glioma.

Parameter	AUC	Cut-off value	Sensitivity	Specificity	Accuracy	Positive predictive value	Negative predictive value
SUV 10	0.807	2.32	87.5%	72.7%	80.4%	77.8%	84.2%
SUV 60	0.762	2.33	91.7%	63.6%	78.3%	73.3%	87.5%
TBR 10	0.826	1.44	100%	63.6%	82.6%	75%	100%
TBR 60	0.821	1.602	95.8%	72.7%	81.8%	79.3%	94.1%
Relative SUV	0.706	1.095	79.2%	63.6%	71.7%	70.4%	73.7%
Relative TBR	0.706	0.914	70.8%	77.3%	73.9%	77.3%	70.8%
SUV difference	0.721	-0.2	58.3%	86.4%	71.7%	82.4%	65.5%
TBR difference	0.774	-0.219	70.8%	90.9%	80.4%	89.5%	74.1%
SUV sum	0.804	4.7	91.7%	72.7%	82.6%	78.6%	88.9%
**TBR sum**	**0.852**	**3.151**	**100%**	**72.7%**	**87%**	**80%**	**100%**

**Fig 1:**
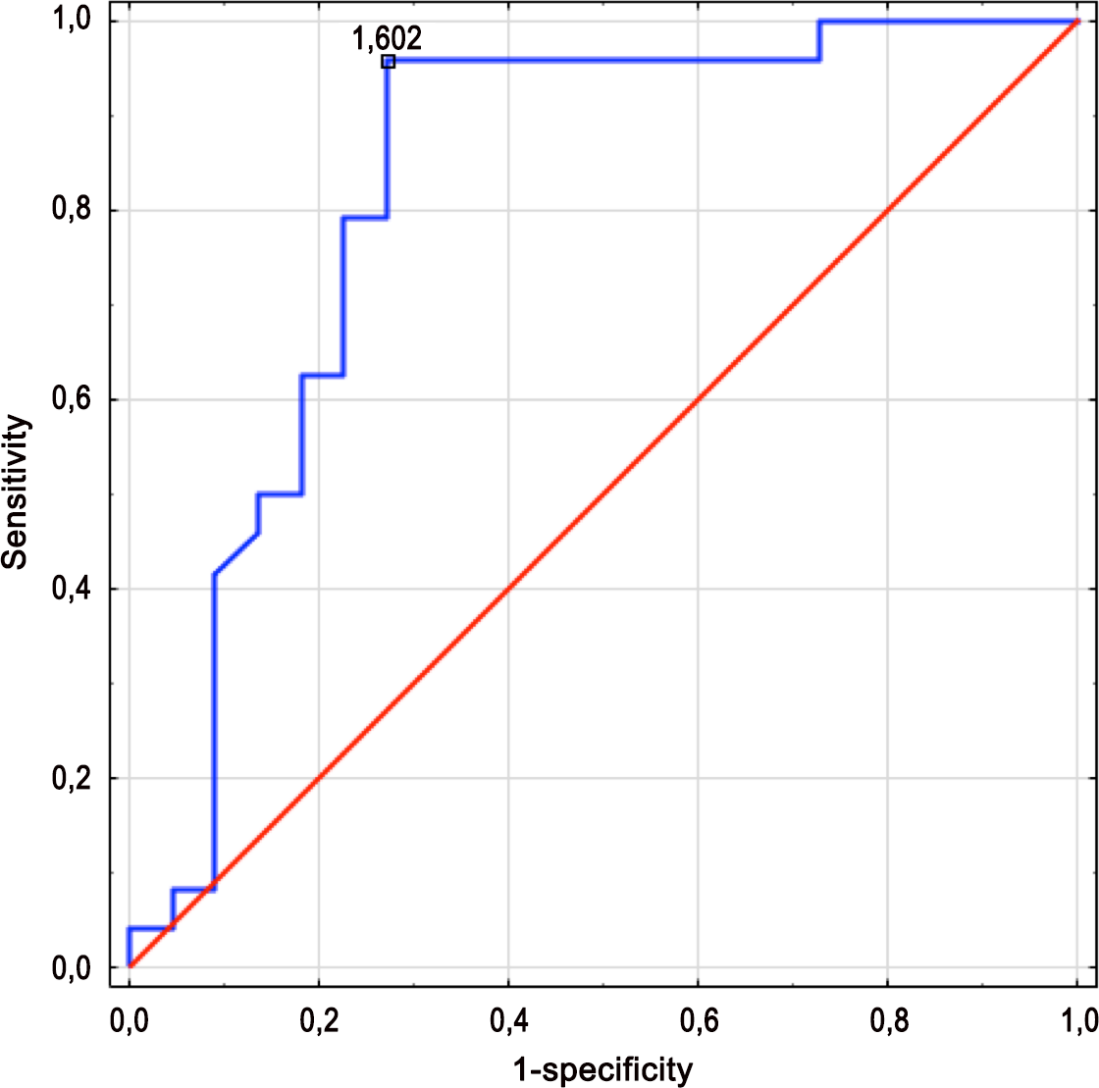
ROC curve for the combined parameter TBR10 + TBR difference; Threshold = 1.602.

Using the ROC curve, we also analysed whether combining the TBR difference with the TBR10 value improved accuracy. This resulted in an AUC of 0.821, sensitivity 95.8%, specificity 72.7%, accuracy 84.8%, and a cut-off value of 1.602 (Fig 1).

**Fig 2:**
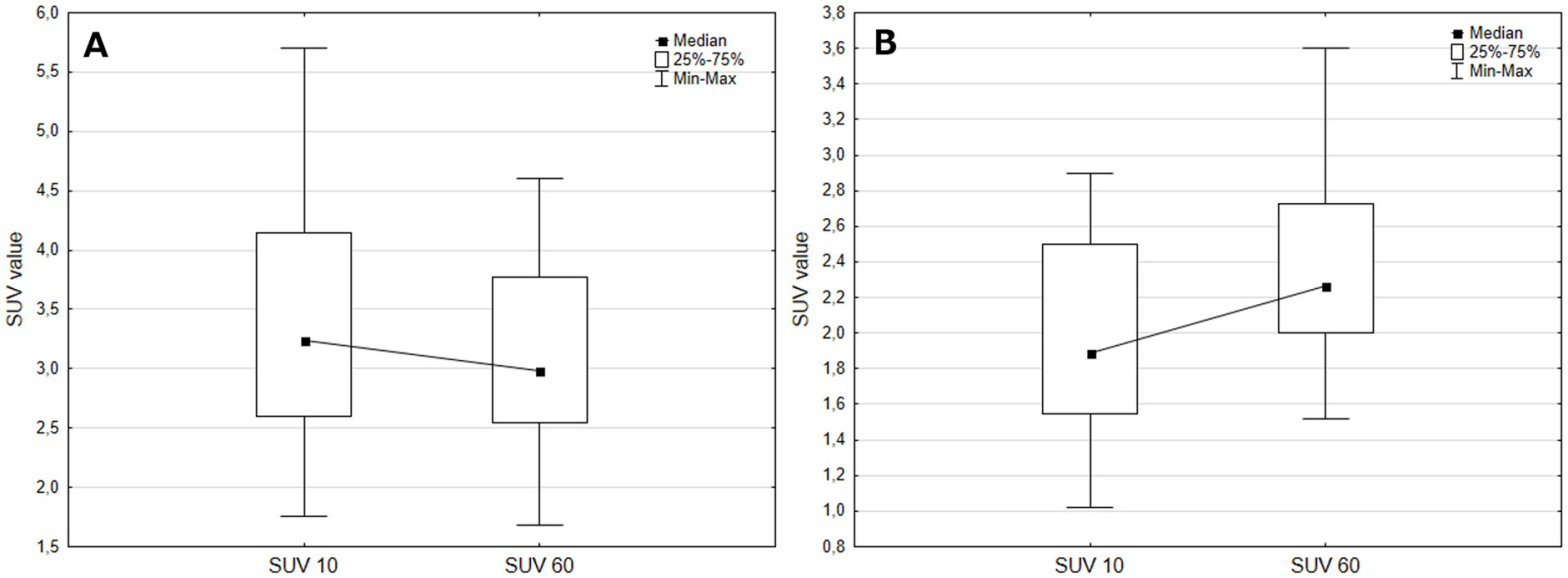
SUV kinetics for high (left graph) and low (right graph) grade gliomas.

The SUV kinetics were different between LGGs and HGGs. The median HGG uptake value decreased and the median LGG uptake value increased on second measurement (Fig 2).

## Discussion

18F-FET PET emerged as a useful diagnostic tool after it was observed that 18-FET undergoes preferential uptake in LGGs and HGGs compared to healthy tissues [11,12]. In glioma patients, preoperative MRI alone has a sensitivity of 68% compared to 97% when MRI is combined with FET PET [7]. PET-based diagnosis of gliomas may be particularly useful when surgery or biopsy are difficult to perform or are not feasible. It has been shown that low 18F-FET uptake in itself is not tumour specific [13], not least because FET uptake is dependent on LAT1 transport system expression [14]. However, there is currently no consensus as to whether a visual or threshold-based approach should be used to define a PET-positive lesion [15]. Here we show that the TBR sum parameter has the greatest accuracy, sensitivity, and negative predictive value for glioma grade differentiation.

We used a threshold method previously described by Pauleit et al. [4] to diagnose gliomas. Using this approach, there was only one false negative case out of the 46 examined, equating to 97% PET sensitivity. These results are consistent with the 92% PET sensitivity observed by Pauleit et al., and are slightly better than those presented in a meta-analysis (82%) examining the use of FET PET to distinguish brain tumours from non-malignant lesions [9]. It should be emphasised that our study included only patients with a strong suspicion of gliomas on MRI, while studies included in the meta-analysis used various criteria for defining FET PET positivity. This might have increased the sensitivity of our study.

The role of single time-point FET PET in the differential diagnosis of gliomas is controversial, with a number of factors known to limit SUV reproducibility [16]. This uncertainty led several investigators to use dynamic SUV changes at different time points from radioisotope administration (Table 4). In the Pauleit et al. study, there was a significant overlap in FET uptake between LGGs and HGGs. Importantly, they measured TBR_max_ up to 30 and 50 min after a radioisotope injection [4]. In our study and in Weckesser et al.’s study, TBR_max_ measured 10 min after 18F-FET injection resulted in an accuracy of between 82.6% and 90% [17].

**Table 4:** Studies estimating TBR_max_ values in gliomas.

Author	No. of tumours of glial origin	Centre/time interval	TBRmax threshold for LG vs. HG tumours, time (min) after FET injection	TBRmax sensitivity/specificity (%)	TBRmax kinetics measurement method	TAC characteristics	TAC sensitivity/specificity
Weckesser 2005 [17]	22	Münster/Jülich Germany before 2004	1.33 10 min	90% accuracy	4x10min intervals from 0–60 min	HGG decrease LGG increase	No data
Stockhammer 2008 [22]	22 No contrast	Berlin Germany	No difference observed 10 min	No data	No data	No data	No data
Popperl 2007 [18]	54	Munich Germany	2.58; sum image: 20–40min	All gliomas 71/85 Astrocytomas 97/73	7 intervals from 0 to 60 min	HGG decrease LGG increase	All gliomas 94/100 Astro-cytomas 94/100
Pauleit 2009 [4]	43	Dusseldorf/ Julich Germany 2004–2005	2.1 (II) vs. 3.7 (III) vs. 3.6 (IV) no threshold data 30–50 min	No data	No data	No data	No data
Hutterer 2013 [13]	131 HGGs 105 LGGs	Innsbruck Austria	2.0; 30 min	No data	No data	No data	No data
**Current study**	**46**	**BydgoszczPoland**	**1.44; 10 min**	**75/100**	**10 min and 60 min p.i.**	**HGG decrease LGG increase**	**70.8/90.9 (TBR difference)**

Using the same methodology as Weckesser et al. to define TBR_max_ resulted in a similar threshold for differentiating LGGs and HGGs. Nevertheless, an inter-institutional calibration procedure would be required to reproduce TBR_max_ and TBR sum cut-off levels between facilities. To limit cut-off value variability, inter-institutional standardisation is required using a calibrated phantom and standardised cross-calibration procedure. Furthermore, standardisation of the injected tracer activity would be desirable.

### Estimation of dual time-point parameters

Taking previously reported 18F-FET glioma uptake kinetics into account [17–19], here we measured SUV and TBR at two time points. HGGs tend to disrupt the blood-brain barrier or blood-tumour barrier more frequently than LGGs, which may facilitate FET transport from the tumour cell back into the vessel and explain altered 18F-FET kinetics in HGGs [17]: high uptake after 10 min and decreasing kinetics between 10 and 60 min after tracer injection is more specific for HGGs. LGGs are usually characterised by different kinetics that increase to reach a maximum 50–60 min after injection. TBR sum defined using dual time-point measurements improves the single time-point results and is simple to define.

In our study, the increase in SUV_max_ and TBR_max_ over time appeared to be characteristic for LGGs (TBR_max_ and SUV_max_ difference > 0, relative TBR_max_ and SUV_max_ > 1), while an inverse kinetics described HGGs (TBR_max_ and SUV_max_ difference < 0, relative TBR_max_ and SUV_max_ < 1). Assessing kinetics using TBR difference and relative TBR is more accurate than similar simple SUV-based parameters, since TBR defines tracer uptake in both the tumour and normal brain. The accuracy is high but limited, which is consistent with Popperl et al [18]. In that study, uptake kinetics was presented as increasing or decreasing the time activity curve (TAC), an approach that was only able to distinguish between WHO I and IV tumours [18]. The authors estimated the sensitivity and specificity of TAC for grade I and grade IV tumours as 94% and 100%, respectively. Using TAC to discriminate between grade II, III, and IV tumours may be limited by the heterogeneous nature of gliomas [19–21]. To analyse the FET uptake dynamics, Popperl et al. also used SUV90 to grade gliomas, which is the mean radioisotope uptake in 90% of the region of interest (tumour area) registered 10 and 60 min after radioisotope injection [19]. They suggested that SUV90 is theoretically unaffected by tumour heterogeneity, but the results did not support TAC as an accurate method to differentiate between WHO II, III, and IV gliomas [[19]; Table 4]. Tumour heterogeneity may alter the uptake kinetics and single time-point acquisition parameters of LGGs and HGGs. Until a method is devised that can quantify heterogeneity, a simple parameter independent of heterogeneity is desirable. The TBR sum parameter takes into account both the uptake value and kinetics and this may be the reason for its accuracy in our study.

TBR difference parameters reflect how the uptake value in gliomatous tissue changes over time. Our study shows that a greater difference can be seen in HGGs. Also, HGG uptake after 10 min was higher than in LGGs, which is consistent with previous reports [17,18]. Combining these features slightly improves the accuracy of each parameter alone, but it fails to improve TBR sum accuracy.

Interestingly, a single time-point measurement tested in a group of gliomas with an intact blood-brain barrier failed to differentiate between high- and low-grade histologies [22]. The biological rationale for a difference in 18F-FET uptake kinetics between LGGs and HGGs with an intact blood-brain or blood-tumour barrier may include tumour angiogenesis, capillary density, or a divergent amount of amino acid transporters in tumour vessels [23]. High 18F-FET uptake in HGGs after 10 min may be facilitated by the high blood supply typical in these tumours [17]. From our perspective, TBR sum could potentially be helpful in a cohort without blood-brain barrier disruption and should be verified in this setting.

Combining MRI perfusion and diffusion images, measuring the relative cerebral blood volume (rCBV), and determining metabolite ratios from proton MR (MRS) spectroscopy increases the accuracy of glioma grading [24]. The threshold value of 1.75 for rCBV provides a sensitivity and specificity of 95.0% and 57.5%, respectively. The combination of rCBV and MRS resulted in a sensitivity and specificity of 93.3% and 60.0%, respectively [25], while the combination of diffusion MRI and dynamic PET revealed improved sensitivity (67–86%) and specificity (63–67% to 100%) [9]. However, even the combined MRI and PET modalities assessed in the latter study were of lower accuracy than TBR sum defined using dynamic PET alone, as reported here. Nevertheless, FET PET and MRI are complementary non-invasive diagnostic modalities in unresectable gliomas.

Our study has some limitations. We studied a relatively small number of patients, in particular for oligodendrogliomas. Hutterer et al. reported that low-grade oligodendrogliomas may produce higher uptake values [13], but our series contained only one patient with oligodendroglioma WHO II (SUV10 = 1.58; SUV60 = 2.19, TBR sum = 2,16). Finally, it should be emphasised that the threshold level of any SUV-based parameter such as TBR sum may change due to PET camera calibration, PET protocols (e.g., a scanning time point and an image reconstruction algorithm), data analysis (e.g., ROI position and size and BG on the contralateral hemisphere or cerebellum), or data settings (maximum and mean), which together may have an effect greater than 50% on the measured parameter [13,26].

## Conclusions

Our study confirms the value of 18F-FET PET in non-invasive determination of glioma grade and supports the value of dual time-point acquisition. The TBR sum parameter shows the greatest accuracy, sensitivity, and negative predictive value for tumour grade differentiation. TBR sum evaluation is a simple method but the cut-off may differ between institutions.
